# Pediatric anesthesia outcomes in Scandinavia: Everything sorted after APRICOT and NECTARINE?

**DOI:** 10.1111/aas.14561

**Published:** 2024-12-23

**Authors:** Tom G. Hansen, Thomas Engelhardt

**Affiliations:** ^1^ Department of Anaesthesiology & Intensive Care Medicine Akershus University Hospital Lørenskog Norway; ^2^ Institute of Clinical Medicine, Oslo University Oslo Norway; ^3^ Department of Pediatric Anesthesia Montreal Children's Hospital Montreal Quebec Canada; ^4^ Department of Anesthesia McGill University Montreal Quebec Canada

The practice of pediatric anesthesia is commonly perceived as risky when compared with adults with a wide variation across healthcare systems. Numerous studies have been published indicating both; higher morbidity and mortality, especially in neonates and young infants. Respiratory and cardiovascular complications regularly account for the vast majority of adverse events.

These poor outcomes were recognized by the Scandinavian Society of Anaesthesia and Intensive Care (SSAI) more than two decades ago. The SSAI advocated for the provision of specialist care for children and centralization in Scandinavian countries. Most importantly, the Scandinavian pediatric anesthesia fellowship was established. The fellowship duration is currently 24 months and comprises 21 months in pediatric anesthesia and a minimum of 3 months in pediatric intensive care. At least 12 months of training must be completed at a highly specialized center affiliated with the fellowship program. Undoubtedly, pediatric and neonatal perioperative care must have improved. But has it really? What is the evidence for this claim? Are we in a position to substantiate this with robust data for the Scandinavian countries?

This is the time to get fruity. Two major European projects have provided some insight into this issue. The Anaesthesia PRactice In Children Observational Trial (APRICOT) and the NEonates and Children audiT of Anaesthesia pRactice IN Europe (NECTARINE) reported detailed prospective pan‐European data on perioperative care.^1^, ^2^ A major strength of both studies was the use of detailed standardized definitions of serious critical events in pediatric and neonatal anesthesia. They provide the most current evidence to inform the successes and failures of different healthcare systems and permit direct comparisons in Europe. The APRICOT study recorded an overall incidence of severe critical events in 1 in 20 children across Europe in more than 31,000 patients. The most dominant recorded events were unsurprisingly attributed to respiratory and cardiovascular problems. The Scandinavian data in more than 1500 patients, or 4% of the cohort, compared favorably in both categories with cardiovascular instability occurring in less than half of the overall reported incidents.^3^ Notably, the proportion of sicker patients where less experienced teams were managing the care was much lower in Scandinavia than in the rest of Europe.

Due to a very small sample size of neonates and young infants, the subsequent NECTARINE study further explored critical events requiring intervention with subsequent 30‐day morbidity and mortality in more than 5000 patients. Critical events occurred in one in three neonates and young infants with hypotension and hypoxia being the most frequently reported events. The triad of hypoxia, hypotension, and anemia, which can be read as compromised tissue oxygen delivery, increases the relative risk of morbidity and mortality to more than 3‐fold and almost 20‐fold at 30 days, respectively. Participating centers across Europe in the NECTARINE study were more homogenous with most being tertiary and quaternary centers caring for the sickest patients. This may have introduced a selection bias and undoubtedly contributed to the reported very high incidence of critical events and the high relative risk of poor outcomes. Here, the results of the Nordic cohort of almost 450 patients, representing 8% of the entire cohort, were nearly identical to the overall NECTARINE cohort. Given the high incidence of critical events, the sample size was insufficient for in depths meaningful comparison.^4^

Both studies elegantly underline that neonates and children in Nordic countries are currently receiving the best care available across Europe and these results need to be considered in a wider perspective. Pediatric healthcare provision differs vastly across European countries as well as requirements in postgraduate medical training.^5^ They are largely attributed to the variability in economic wealth, although this must not be used as an excuse to accept or deliver poor perioperative care. In Scandinavia, there is a perceived luxury of always two anesthesia‐trained professionals being present at the induction and emergence of anesthesia. In addition, in the post‐anesthesia care units, children are recovered by trained and registered specialist pediatric nurses. Despite the centralization of surgical and anesthesia care for neonates and infants at tertiary pediatric as well as older children with moderate or severe co‐morbidities, some infants still undergo general anesthesia in county and district hospitals that are not reflected in the APRICOT or NECTARINE studies. These are not captured robustly in either study.

The successful outcome data in Scandinavia are no justification to ease our efforts to improve training in pediatric anesthesia and educational infrastructure in the Nordic countries. This requires continuing efforts. Each Nordic National Professional Society of Anesthesia and Intensive Care has a subsection for pediatric anesthesia. They organize annual scientific meetings as well as refresher courses in pediatric anesthesia. While this is a significant milestone in pediatric anesthesia in the Nordic countries, pediatric anesthesia is still not an officially recognized anesthesia subspecialty. We believe that the SSAI and the national societies should embrace this notion and rectify this issue to maintain the apparent current advantage in health care provision in this patient population.

The focus on specialized training and centralization of pediatric anesthesia in Scandinavia has undoubtedly led to improvements in patient safety and outcomes for most children undergoing anesthesia, as evidenced by the APRICOT and NECTARINE data. However, we need to recognize also a notable decline in the experience and competence of generalist anesthesiologists when tasked to look even after the most simple pediatric cases. This may potentially compromise timely care and adversely affect outcomes. The impact may be most severe in more remote areas or children requiring emergency care. This highlights the need for additional strategies and resources that balance specialization while maintaining competence in pediatric anesthesia in non‐specialist anesthesia practitioners. We hope that the SSAI and the National Societies will support and promote this critical step forward.

Clinical practices need to be examined as to their merit. The existing pediatric anesthesia practices in Scandinavia may be more uniform when compared to the rest of Europe.^5^ However, analyzing and reporting only sporadically perioperative outcome data may be insufficient to maintain or indeed improve care for young children and neonates. Best practice protocols and successful quality improvement initiatives must be shared and clinical outcome surveillance databases established. The reported reproducible critical event definitions can be used as a starting point. Long‐term, patient‐reported outcome data are still sparse in children undergoing a wide variety of surgical or diagnostic procedures. Limiting ourselves to studying only up to 30 or 60 days morbidity and mortality is short‐sighted with the latter exceedingly rare in most anesthesia‐reported outcome reports. Future clinical trials should be guided under the umbrella of The Core Outcome Measures in Effectiveness Trials (COMET) initiative (https://www.comet-initiative.org/). While this is well developed in adults with defined outcomes such as perioperative myocardial infarction, stroke, cognitive decline, or even return to work, such outcomes remain less defined in children. Within the SSAI, we should strive to develop a close investigative collaboration and input with the Pediatric Perioperative Outcomes Group ensuring that the Scandinavian voice is heard.

While the Scandinavian and Nordic data of the APRICOT and NECTARINE study shed a favorable light on the perioperative pediatric anesthesia practice, we are mandated to search for continuing improvement. Only future reports will confirm if we are still heading in the right direction.

## Data Availability

Data sharing is not applicable to this article as no new data were created or analyzed in this study.
